# Contrast enhancement by location and volume is associated with long-term outcome after thrombectomy in acute ischemic stroke

**DOI:** 10.1038/s41598-022-21276-3

**Published:** 2022-10-10

**Authors:** Guo-Can Chang, Dai-Chao Ma, Wei Li, Jin Qiu, Xian-Hui Sun, Yong-Gang Zhao, Xin Liu, Zi-Ai Zhao, Liang Liu, Thanh N. Nguyen, Hui-Sheng Chen

**Affiliations:** 1 Department of Neurology, General Hospital of Northern Theater Command, Shen Yang, People’s Republic of China; 2 Neurology, Radiology, Boston Medical Center, Boston, MA USA; 3 Present Address: Department of Encephalopathy, Affiliated Hospital of Shaanxi University of Chinese Medicine, Xianyang, People’s Republic of China

**Keywords:** Stroke, Outcomes research

## Abstract

Contrast enhancement (CE) on brain non-contrast computed tomography (NCCT) is common after endovascular thrombectomy (EVT) in patients with acute ischemic stroke (AIS), but its association with clinical outcomes is not well established. The current study aimed to investigate this relationship. We retrospectively reviewed consecutive patients with acute ischemic stroke who had hyperdensity on NCCT immediately after EVT for anterior circulation large vessel occlusion (LVO) from January 2016 to December 2019. We used ASPECTS combined with volume measurement by 3D reconstruction to estimate the extent and location of CE. Multivariable regression analysis was conducted to explore the risk factors associated with clinical outcome. In this study, 113 of 158 (71.52%) anterior circulation AIS-LVO patients had hyperdensity on brain NCCT. After strict inclusion and exclusion criteria, a total of 64 patients were enrolled in the final analysis. In logistic regression analysis, CE-ASPECTS, CE volume, CE at the caudate nucleus, M4 and M6 region were associated with 3-month poor functional outcome after adjusting for confounding factors. The conventional variable model was used for reference, including age, initial NIHSS, the procedure time, stent retriever passes, recanalization status and baseline ASPECTS, with AUC of 0.73. When combined with the above-named variables (conventional variables + CE-ASPECTS + CE volume + CE at caudate nucleus + CE at M4 region + CE at M6 region), the predictive power was significantly improved, with AUC of 0.87 (95% CI 0.78–0.95). The spatial location and volume of CE on NCCT obtained immediately after EVT were independent and strong predictors for poor outcome at 3-months in patients with AIS after excluding definite hemorrhage by 24-h follow up CT.

## Introduction

Endovascular thrombectomy (EVT) is recognized to be an effective therapeutic strategy to restore blood flow in acute ischemic stroke (AIS) due to large vessel occlusion (LVO)^1,2^. Contrast enhancement (CE), defined as a visually distinct hyper-density on non-contrast computed tomography (NCCT) obtained immediately after the procedure, is commonly found with a reported incidence of 30.7–87.5%^3–10^. CE is believed to be due to disruption of the blood brain barrier (BBB)^4,11–13^, resulting in extravascular leakage of contrast medium, some of which may be mixed with extravascular blood.

Some studies have reported that the presence of CE was a risk factor of functional outcomes^3–5^, while others did not find prognostic value^8–10^. We argue that the controversial results may be due to heterogeneous definitions of hyperdensity, the different computed tomography equipment used after EVT, or the lack of quantitative assessment of CE. The prognostic value of hyperdensity on NCCT after EVT has not been well established and merits further investigation.

The Alberta Stroke Program Early CT Score (ASPECTS), a quantitative topographic scoring system, divides the MCA territory into 10 regions based on functional importance rather than extent, which was a simple and reliable systematic method to assess early ischemic change^14,15^. We hypothesized that this localization weighted method of ASPECTS is also suitable for evaluating the severity of CE. Based on the spatial distribution of CE referring to the ASPECTS system and its volumes on NCCT immediately after EVT, the present study aimed to investigate the relationship between CE and clinical outcome in patients with AIS.

## Methods

### Patients

This retrospective study was approved by institutional review board of General Hospital of Northern Theater Command (IRB: Y(2020)087) with waiver of informed consent. All methods were carried out in accordance with relevant guidelines and regulations. Between June 2016 and October 2019, we retrospectively screened consecutive patients with EVT from the Department of Neurology of the General Hospital of Northern Theater Command. Electronic medical records and imaging information were obtained by the hospital information system, and picture archiving and communication systems connected by a local area network. The inclusion criteria were as follows: (1) Patients with acute anterior circulation ischemic stroke with LVO who underwent EVT within 24 h of onset; (2) Prestroke modified Rankin Scale (mRS) score was less than or equal to 1; (3) There was hyperdensity on the NECT within 1 h after EVT, which was difficult to distinguish between the contrast agent and hemorrhage by two experienced neurointerventionists (J.Q and W. L). Patients were excluded for the following reasons: (1) patients who did not receive NCCT within 1 h after thrombectomy or failed to follow-up; (2) definite hemorrhagic transformation on the 24-h follow-up NCCT; (3) inadequate clinical data; (4) unqualified image due to extensive artifacts.

### Data collection

The baseline characteristics were collected from the medical records, including demographics, medical history and risk factors of cerebrovascular diseases (smoking, alcohol drinking, hypertension, diabetes mellitus, and atrial fibrillation), stroke characteristics, pharmacological therapies, neuroimaging features, and procedural details. The patient’s stroke severity on admission was assessed by the National Institute of Health Stroke Scale (NIHSS).

### Data availability

The data underlying this article will be shared upon reasonable request to the corresponding author.

### Imaging analysis

**Figure 1:**
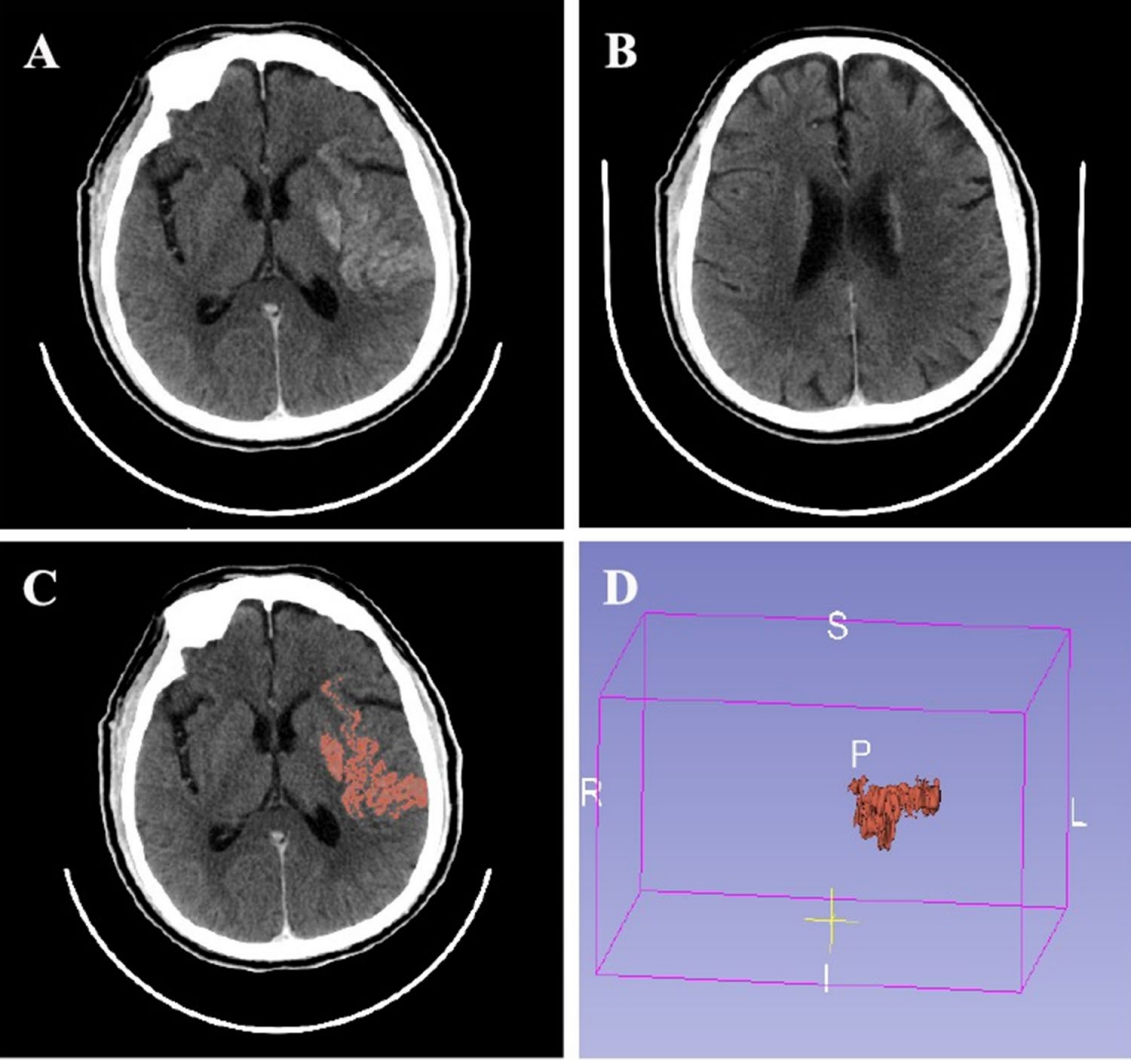
A 71-year-old patient presented with left-sided weakness (CE-ASPECT 6 and CE-Volume 9.041 ml). (**A**) and (**B**) Showing CE in the insula, lenticular nucleus, M1 and M2 ASPECTS regions. Thus, the CE-ASPECTS was counted as 6. (**C**) and (**D**) The three-dimensional structure of CE in the brain was reconstructed with a volume of 9.041 ml.

Non-contrast cranial CT (120 kV, mA/slice 400 mA, 5.0 mm axial images, the field of view 25 cm; General Electric, Boston, United States of America) was used in this study. ASPECTS was utilized to assess the spatial distribution of CE, and allotted 10 points of the territory of the middle cerebral artery. Each ASPECTS region was scored as 0 if CE was present, as 1 if there was no CE^14^. CE -ASPECTS and any location of CE was evaluated and recorded (Fig. 1A,B).

The CE volume was measured by automated 3D-Slicer software (Fig. 1C,D). The CT data of the patient in Digital Imaging and Communications in Medicine (DICOM) format was imported into the 3D Slicer software (3D Slicer 4.11). After adjusting the window width and window level to CT-brain, the next steps were as follows: run Editor → Threshold → Use for masking. By using the threshold range that was manually set according to the HU value of CE for masking and switching to paint effect, the pixels in which CE was present was further manually marked. Given the possible intersection of threshold ranges between normal brain tissue and CE, the individual threshold range (the maximum and minimum HU values) was set according to the HU value of CE in each patient based on Image J software to assure the reliability and reproducibility of the measurement method. Then, the three-dimensional reconstruction of the CE was realized, and the volume of the CE was calculated automatically^16^.

Hemorrhage was defined as a persistent intraparenchymal hyperdense area on the follow-up CT which did not resolve for more than 24 h after EVT.

All images were independently evaluated by two experienced investigators (J.Q and W. L) who were blinded to the clinical data. Disagreements between these investigators were reviewed by a third adjudicator (Z.A.Z), and were resolved by majority opinion.

### Main outcomes

The primary outcomes of the study were to evaluate good and poor outcome, which was defined as mRS 0–2 and mRS 3–6 at 90 days after stroke, respectively.

### Measurement reproducibility

To assess intrarater and interrater variability, two experienced investigators (J.Q and W. L) who were blind to the clinical data independently reassessed CE volume and CE -ASPECT of all patients 1 month later.

### Statistical analysis

Continuous variables are expressed as the mean with SD, or median with interquartile range (IQR), and categorical variables were described as frequencies and percentages. Continuous variables were compared with the t-test or Mann–Whitney U test. The chi-square test or Fisher’s exact test was used to compare categorical variables. Multivariable analysis was performed using multiple logistic regression analysis to evaluate factors that may predict progression to poor outcome. We adjusted for the clinical outcome-related covariates reported in previous studies, including age, initial NIHSS, the procedure time, number of stent retriever passes and mTICI 2b-3. Multicollinearity of the independent variables were analyzed using the variance inflation factor (VIF) statistic. Interrater and intrarater variability of CE volume and CE-ASPECTS was tested with intraclass correlation coefficient (ICC) (two-way random, absolute agreement, single measures). All statistical analyses were conducted using IBM SPSS version 25.0 (IBM Inc), and a *P* value less than 0.05 was considered statistically significant.

### Ethics approval

This retrospective study was approved by our institutional review board (IRB: Y (2020)087).

## Results

**Figure 2:**
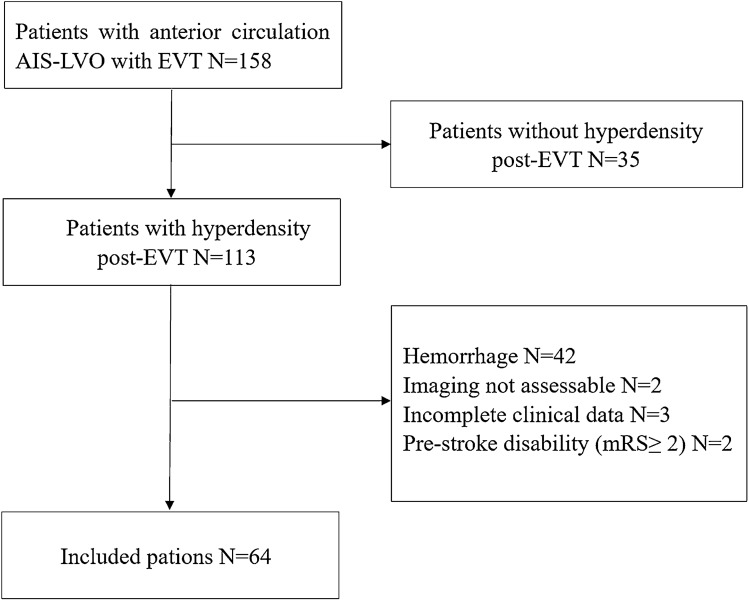
A flowchart of subject selection.

We retrospectively included 158 consecutive anterior circulation AIS-LVO patients of which 113 (71.52%) had hyperdensity on NCCT within 1 h after EVT. We excluded patients with hemorrhage (n = 42), imaging not assessable (n = 2), incomplete clinical data (n = 3) and pre-stroke disability (mRS ≥ 2) (n = 2). Finally, the remaining 64 patients were enrolled for the current analysis (Fig. 2).

The baseline characteristics of the patients are demonstrated in Table 1. A total of 28 patients (44%) had good outcome and 36 patients (56%) had poor outcome. Patients with good clinical outcome had lower median initial NIHSS score (13 (± 4) vs. 15 (± 5); *P* = 0.046), higher CE-ASPECTS score (7 (±2) vs. 5 (±3); *P* = 0.031) and lower CE volume (5.89 (±8.51) vs. 33.35 (±65.16); *P* = 0.015). Higher prevalence of CE at M2, M4, M5, and M6 regions were noted in the poor outcome group (all *P* < 0.05). There were no significant differences in other factors between the two groups.

**Table 1:** Baseline characteristics of 64 patients.

Characteristic	Good outcome	Poor outcome	*P* value
	(n = 28)	(n = 36)	
Age, mean (SD), y	62.0 (10.6)	65.0 (9.8)	0.241
Female, n (%)	11 (39.3)	9 (25.0)	0.221
SBP, mean (SD), mmHg	162.6 (24.9)	160.55 (24.4)	0.735
DBP, mean (SD), mmHg	93.3 (17.9)	90.7 (13.9)	0.522
Smoking, n (%)	18 (64.3)	18 (50.0)	0.253
Alcohol drinking, n (%)	18 (64.3)	17 (17.2)	0.174
Hypertension, n (%)	21 (72.0)	20 (55.6)	0.108
Diabetes, n (%)	5 (17.9)	7 (19.4)	0.872
Atrial fibrillation, n (%)	9 (32.1)	10 (27.8)	0.705
Serum creatinine	64.6 (57.4, 88.7)	74.0 (62.4, 94.7)	0.223
**TOAST classification**			0.908
LAA, n (%)	18 (64.3)	24 (66.7)	
Cardioembolism, n (%)	5 (17.9)	7 (19.4)	
Other, n (%)	5 (17.9)	5 (13.9)	
Initial NIHSS, mean (SD)	13 (4)	15 (5)	0.046
Baseline ASPECTS, median (IQR)	8.5 (8, 9)	9 (7, 9)	0.878
Intravenous thrombolysis, n (%)	10 (35.7)	13 (36.1)	0.974
OPT, median (IQR), min	413.50 (294.75, 733.50)	398.50 (300.00, 622.50)	0.720
OET, median (IQR), min	572.50 (408.50, 815.50)	505.00 (392.50, 771.00)	0.908
The procedure time, median (IQR), min	90.5 (62.8, 150.5)	97.50 (82.0, 175.0)	0.147
Passes of retriever, median (IQR)	1 (1, 2)	1 (1, 1)	0.304
mTICI 2b-3, n (%)	17 (60.7)	16 (44.4)	0.196
CE-ASPECTS, mean (SD)	7 (2)	5 (3)	0.031
CE volume, mean (SD), ml	5.89 (8.51)	33.35 (65.16)	0.015
**CE locations**			
C, n (%)	2 (7.1)	9 (25.0)	0.095
IC, n (%)	5 (17.9)	7 (19.4)	0.872
L, n (%)	22 (78.2)	28 (77.8)	0.939
I, n (%)	13 (46.4)	22 (61.1)	0.242
M1, n (%)	9 (32.1)	19 (52.8)	0.099
M2, n (%)	9 (32.1)	21 (58.3)	0.037
M3, n (%)	7 (25.0)	15 (41.7)	0.164
M4, n (%)	5 (17.9)	18 (50.0)	0.008
M5, n (%)	9 (32.1)	21 (58.3)	0.037
M6, n (%)	6 (21.4)	18 (50.0)	0.019

*SBP* systolic blood pressure, *DBP* diastolic blood pressure, *LAA* large artery atherosclerosis, *SD* standard deviation, *NIHSS* National Institutes of Health Stroke Scale, *ASPECTS* Alberta stroke program early computed tomography score, *IQR* interquartile range, *OPT* onset-to-procedure time, *OET* onset to the end of EVT time, *mTICI* modified Thrombolysis in Cerebral Infarction, *CE* contrast enhancement, *C* caudate nucleus, *L* lentiform, *IC* internal capsule, *I* insular ribbon.

On univariable analysis, lower CE-ASPECTS score and higher CE volume were significantly associated with poor clinical outcome after EVT. Additionally, the presence of CE at the caudate nucleus, M2, M4, M5, and M6 region was also found to be related to poor outcome. Higher initial NIHSS score showed a tendency to poor prognosis (*P* = 0.051), but other factors failed to show a significant association (Table 2). In addition, the scores of CE-ASPECTS was associated with baseline ASPECTS of infarct lesions (ρ = 0.31; *P* < 0.05, Spearman correlation test).

**Table 2:** Univariable logistic regression analysis of predictors for poor outcome.

	OR (95% CI)	*P* value
Age	1.031 (0.980, 1.083)	0.239
Initial NIHSS	1.137 (0.999, 1.293)	0.051
Baseline ASPECTS	1.009 (0.724, 1.406)	0.958
Intravenous thrombolysis	1.017 (0.363, 2.849)	0.974
the procedure time	1.004 (0.999, 1.008)	0.131
Passes of retriever	1.510 (0.726, 3.141)	0.270
mTICI 2b-3	0.518 (0.190, 1.412)	0.198
CE-ASPECTS	0.813 (0.684, 0.966)	0.019
**CE locations**		
C	4.333 (0.854, 21.986)	0.077
M1	2.359 (0.844, 6.597)	0.102
M2	2.956 (1.052, 8.307)	0.040
M4	4.600 (1.432, 14.778)	0.010
M5	2.956 (1.052, 8.307)	0.040
M6	3.667 (1.203, 11.174)	0.022
CE volume (5 ml)	1.283 (1.045, 1.576)	0.017

*NIHSS* National Institutes of Health Stroke Scale, *CE* contrast enhancement, *ASPECTS* Alberta stroke program early computed tomography score, *C* caudate nucleus, *mTICI* modified Thrombolysis in Cerebral Infarction, *95% CI* indicates 95% confidence interval, *OR* odds ratio.

On multivariable logistic regression analysis, CE-ASPECTS (aOR 0.80, 95% CI 0.65–0.98, *P* < 0.05), CE volume (aOR 1.27, 95% CI 1.01–1.61, *P* < 0.05, for every 5 ml increment in CE volume), CE at the caudate nucleus (aOR7.56, 95% CI 1.22–46.82, *P* < 0.05), CE at M4 region (aOR 6.09, 95% CI 1.55–23.99, *P* < 0.05), and M6 region (aOR 4.46, 95% CI 1.19–16.67, *P* < 0.05) were associated with 3-month poor functional outcome after adjusting for age, initial NIHSS, the procedure time, stent retriever passes and recanalization status (mTICI 2b-3) (Table 3). The VIF and tolerance were < 10 and > 0.1, respectively, suggesting no co-linearity among the final predictors (Supplementary Table I).

**Table 3:** Multivariable logistic regression analysis of predictors for poor outcome.

	Adjusted OR (95% CI)	*P* value
CE-ASPECTS	0.798 (0.654, 0.975)	0.027
**CE locations**		
C	7.556 (1.219, 46.822)	0.030
M1	2.227 (0.662, 7.490)	0.196
M2	2.482 (0.780, 7.900)	0.124
M4	6.089 (1.546, 23.988)	0.010
M5	3.255 (0.945, 11.208)	0.061
M6	4.460 (1.193, 16.671)	0.026
CE volume (5 ml)	1.274 (1.011, 1.607)	0.040

Adjusted for age, initial NIHSS, the procedure time, passes of retriever and mTICI 2b-3.

*CE* contrast enhancement, *ASPECTS* Alberta stroke program early computed tomography score, *C* caudate nucleus, *95% CI* indicates 95% confidence interval, *OR* odds ratio.

**Figure 3:**
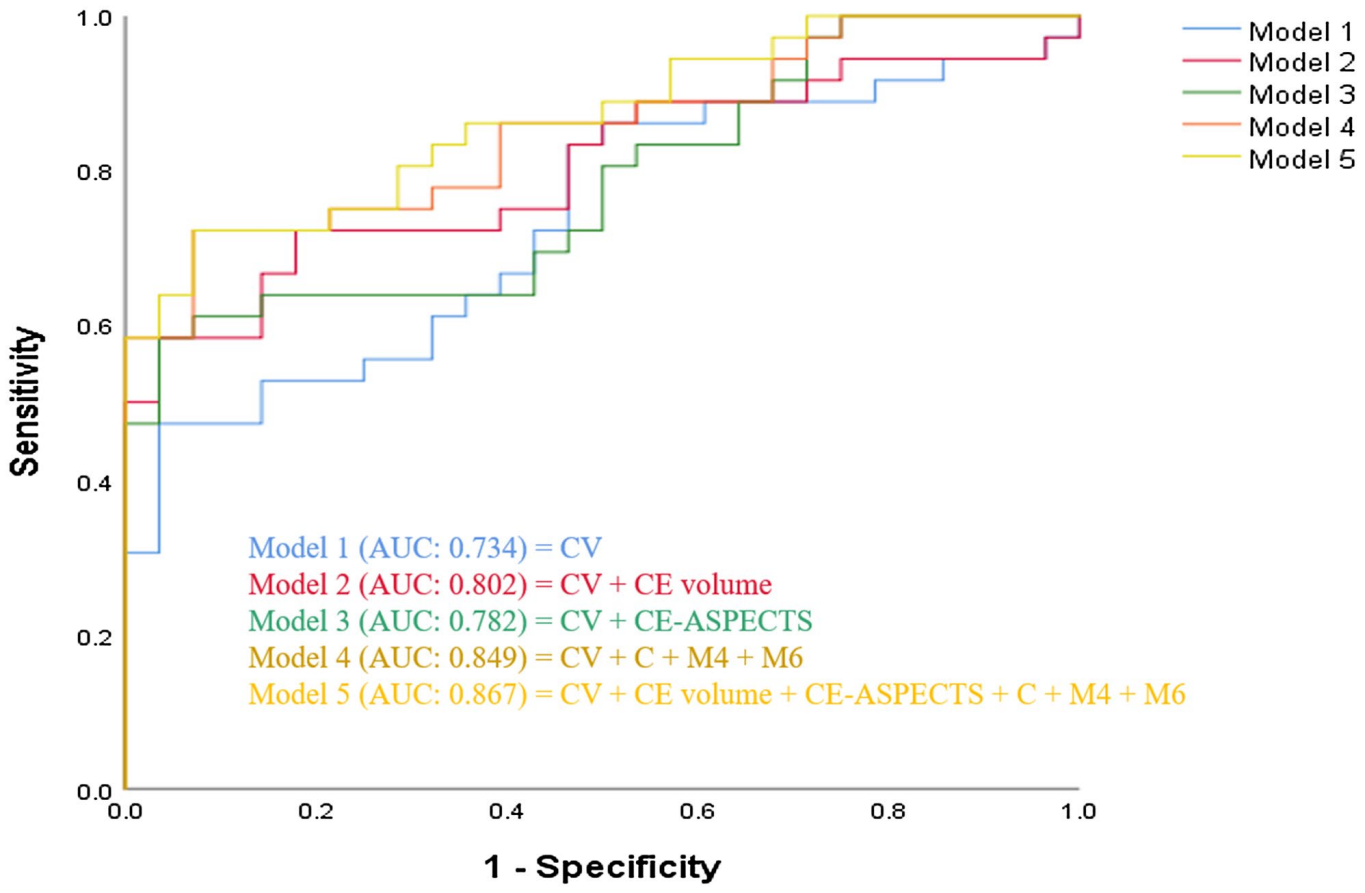
ROC curves comparing the potential of different models to predict poor outcome. CV, conventional variables including age, initial NIHSS, the procedure time, passes of retriever, recanalization status (mTICI 2b-3) and baseline ASPECTS.

As shown in Fig. 3, we built several models to predict poor outcome. First, the conventional variables were used as model 1 for reference, including age, initial NIHSS, the procedure time, stent retriever passes, recanalization status and baseline ASPECTS, with the area under the receiver operator characteristic curve (AUC) of 0.73 (95% CI 0.61–0.86). When individually added to the predictive model based on conventional variables, CE volume (AUC 0.80, 95% CI 0.69–0.91), or CE-ASPECTS (AUC 0.78, 95% CI 0.67–0.89) improved discriminative performance. The location variables of CE, combination of CE at C, M4 and M6 regions, also had significant discriminative performance with an AUC of 0.85 (95%CI 0.76–0.94). When combined with the above-named variables (conventional variables + CE-ASPECTS + CE volume + CE at caudate nucleus + CE at M4 region + CE at M6 region), the predictive power was further improved, with the AUC of 0.87 (95% CI 0.78–0.95), significantly higher than model 1 (∆AUC of 0.13, *p* = 0.014).

Interrater reliability was excellent for both CE volume (ICC = 0.993; 95%CI 0.981–0.996; *p* < 0.001) and CE-ASPECTS (ICC = 0.995; 95%CI 0.991–0.997; *p* < 0.001). Intrarater ICC ranged from 0.993 (95%CI 0.987–0.997) to 0.994 (95%CI 0.988–0.996) for CE volume, and from 0.994 (95%CI 0.993–0.997) to 0.996 (95%CI 0.990–0.996) for CE-ASPECTS (Supplementary Table II).

## Discussion

CE has been commonly found on the NCCT obtained immediately after EVT in patients with AIS^3–10^, which has been attributed to the disruption of the blood brain barrier^4,11–13^. In the present study, the incidence of CE on NCCT was 71.5%, which was comparable to prior reports (30.7–87.5%^3–10^). Our work is novel in that we adapted the methodology of ASPECTS score to CE combined with volume measurement to assess the extent and location of CE, and found that CE based on this quantitative method after EVT was an independent and strong predictor of clinical functional outcome after excluding definite hemorrhage by 24-h follow up CT.

Previous studies have investigated the relationship of the hyperdensity on NCCT and clinical outcomes, but the results were conflicting. Some studies found that the presence of a high-density lesion on NCCT had no prognostic value to clinical outcome^8–10^. In contrast, Portela et al.^3^ found that the total size of the hyperdense area had a positive correlation with 90-day mRS after EVT. As previously reported, the hyperdensity may be related to the contrast agent, hemorrhagic transformation or a combination^9,11,13,17^, which may be caused by varying degrees of BBB disruption. Based on the dual-energy CT (DECT) technology, which allows an accurate differentiation between contrast staining and hemorrhage, Renú et al.^4^ and Chen et al.^5^ reported that contrast staining within 24 h of EVT was associated with an increased risk of hemorrhage and was also an independent predictor of unfavorable clinical outcomes. The definitions of the hyperdensity in these studies were of two kinds: high attenuation and contrast staining, depending on whether it contains hemorrhage. We surmise that the controversial results of these studies may be related to the following reasons: the heterogeneous definitions of hyperdensity based on the different computed tomography techniques after EVT (high attenuation on NCCT^3,8,9^ vs. contrast staining on DECT^4,5,10^), or the lack of quantitative assessment (quantitative^3^ vs. non-quantitative^4,5,8–10^).

We adapted the methodology of ASPECTS score to CE combined with volume measurement by 3D reconstruction to ensure precise evaluation of the extent and location of CE on the NCCT obtained immediately after EVT. To our knowledge, this is the first report to describe this technique in the evaluation of CE after EVT. Although DECT is considered the gold standard of differentiating extravasation of contrast agent and cerebral hemorrhage after EVT, with high sensitivity and specificity^4,5,18–20^, it is not readily available in most stroke centres. In addition, given that hemorrhagic transformation was found to be associated with poor functional outcome in AIS-LVO patients in previous studies, especially PH2 (> 30% of the infarcted area with significant space-occupying effect)^21–23^, we explored the relationship of CE on NCCT with functional outcome after exclusion of cases with definite hemorrhagic transformation. Interestingly, the presence of CE in the caudate, M2, M4, M5, and M6 region were significantly related to poor outcome, which may be explained by the fact that these cortical regions of ASPECTS contained eloquent cerebral functional areas involving the pre-motor, motor and parietal cortex. Another possible explanation is that the presence of CE in cortical areas may be associated with poor collateral circulation, which was an independent predictor of poor clinical prognosis in acute ischemic stroke^24–28^. The other possibility may be associated with pre-existing early infarct prior to EVT, which was supported by the correlation between the scores of CE-ASPECTS and initial ASPECTS of infarct lesions in this study (ρ = 0.31; *P* < 0.05, Spearman correlation test). Of note, the head of the caudate nucleus manifested similar outcomes to the cortex structures, which could be explained by its shared supply from the anterior cerebral artery, the latter territory of which is often eloquent and not included in ASPECTS grading^13,16,29^.

### Limitations

We acknowledge limitations in our study. A main limitation is that a 24-h follow-up CT after EVT is needed to exclude the hemorrhage in the current study, which will limit the clinical value of current findings. However, the current findings will be helpful for the patients whose contrast enhancement after EVT can be evaluated without the need for 24-h CT after EVT. On the other hand, the current findings will provide the important information for patients with contrast enhancement after EVT whose hemorrhage was excluded by early DECT. Second, the data based on a relatively small sample size and single medical center, were susceptible to selection bias. A larger, patient sample may further refine the model. Because of the retrospective nature of the present study, the potential confounders could not be completely controlled. Third, the dose of the contrast agent was not collected in this study due to the retrospective design. It was reported that high amounts of contrast agent could be a potential contributor to BBB disruption due to its toxicity^5,11,30^, and may lead to the neurological complications of contrast-induced encephalopathy^30,31^. In the current study, comparable procedure time and stent retriever passes between groups may indirectly exclude the effect of CE amount. Fourth, we did not record the number of microcatheter injection runs obtained, which is also known to be associated with contrast extravasation and intracranial hemorrhage^32^. Finally, given that the present study focused on the effect of CE on clinical outcome, the patients with definite hemorrhage on the immediate NCCT after EVT were excluded to avoid the confounding effect, thus our conclusion does not apply to patients with hemorrhagic transformation.

## Conclusion

CE-ASPECTS and higher CE volume on NCCT obtained immediately after EVT in patients with AIS may be independently associated with poor functional outcome at 3-months after excluding definite hemorrhage by 24-h follow up CT. The predictive performance for poor outcome was higher when CE was located in the head of caudate nucleus, and the cortical M4 and M6 regions of ASPECTS.

## Supplementary Information

Supplementary Tables.

## Data Availability

Data are available upon reasonable request.
