# Supplementary material for: Contrast enhancement by location and volume is associated with long-term outcome after thrombectomy in acute ischemic stroke
Source: Sci Rep. 2022 Oct 10;12:16998. doi: 10.1038/s41598-022-21276-3 (PMC9551090; doi:10.1038/s41598-022-21276-3)
Supplement: Supplementary file 1 — Supplementary Tables. [file 41598_2022_21276_MOESM1_ESM.docx]

**Supplemental Table I.** VIF and tolerance values of predictors for poor outcome

|  | VIF | tolerance |
| --- | --- | --- |
| C | 1.554 | 0.644 |
| M4 | 3.271 | 0.306 |
| M6 | 2.293 | 0.436 |
| CE volume | 1.409 | 0.710 |
| CE-ASPECTS | 6.895 | 0.145 |

Abbreviation: VIF, variance inflation factor; C, Caudate nucleus; CE, Contrast enhancement; ASPECTS, Alberta stroke program early computed tomography score.

**Supplemental Table II.** Interrater and intrarater reliability of CE volume and CE-ASPECTS

|  | CCI | 95%CI | *P* |
| --- | --- | --- | --- |
| Interrater reliability |  |  |  |
| CE volume | 0.993 | 0.981-0.996 | 0.000 |
| CE-ASPECTS  Intarrater reliability | 0.995 | 0.991-0.997 | 0.000 |
| CE volume |  |  |  |
| I1 | 0.994 | 0.987-0.997 | 0.000 |
| I2 | 0.993 | 0.988-0.996 | 0.000 |
| CE-ASPECTS |  |  |  |
| I2 | 0.996 | 0.993-0.997 | 0.000 |
| I2 | 0.994 | 0.990-0.996 | 0.000 |

Abbreviation: CE, Contrast enhancement; ASPECTS, Alberta stroke program early computed tomography score; ICC, intraclass correlation coefficient; 95% CI, 95% confidence interval; I1, investigator 1; I2, investigator 2.
